# Parameters Associated With Renal Recovery and Survival in Myeloma Patients With Acute Renal Failure to Cast Nephropathy

**DOI:** 10.1002/ajh.70104

**Published:** 2025-10-24

**Authors:** Heinz Ludwig, Meletios Athanasios Dimopoulos, Evangelos Terpos, Sarah Bernhard, Foteini Theodorakakou, Meral Beksac, Guldane Cengiz‐Seval, Nelson Leung, Luca Arcaini, Silvia Mangiacavalli, Frank Bridoux, Hermine Agis, Aristeidis Chaidos, Francesca Gay, Dario Roccatello, Wolfgang Hilbe, Andrea Havasi, Daniele Derudas, Nattawat Klomjit, Kenar D. Jhaveri, Javier De La Rubia Comos, Efstathios Kastritis

**Affiliations:** ^1^ Wilhelminen Cancer Research Institute, c/o Department of Medicine I Clinic Ottakring Vienna Austria; ^2^ Department of Clinical Therapeutics, School of Medicine National and Kapodistrian University of Athens Athens Greece; ^3^ Korea University Seoul South Korea; ^4^ Department of Hematology Ankara University School of Medicine Ankara Turkey; ^5^ Division of Nephrology and Hypertension, Hematology Mayo Clinic Rochester Minnesota USA; ^6^ Department of Molecular Medicine University of Pavia Pavia Italy; ^7^ Division of Hematology Fondazione IRCCS Policlinic San Matteo Pavia Italy; ^8^ Department of Nephrology & Renal Transplantation Université de Poitiers, Centre Hospitalier Universitaire Poitiers France; ^9^ Department of Medicine I, Division Hematology and Hemostaseology Medical University Vienna Vienna Austria; ^10^ Hugh and Josseline Langmuir Centre for Myeloma Research, Centre for Haematology, Department of Immunology and Inflammation Imperial College London and Hammersmith Hospital, Imperial College Healthcare NHS Trust London UK; ^11^ Myeloma Unit, Division of Hematology University of Torino, Azienda Ospedaliero‐Universitaria Città della Salute e della Scienza di Torino Torino Italy; ^12^ ASL Città di Torino and Department of Clinical and Biological Sciences of the University of Turin San Giovanni Bosco Hub Hospital Turin Italy; ^13^ Department of Medicine I, Center for Oncology and Hematology Clinic Ottakring Vienna Austria; ^14^ Renal Section, Boston University School of Medicine Boston Massachusetts USA; ^15^ Department of Hematology Businco Hospital Cagliari Italy; ^16^ Division of Nephrology and Hypertension University of Minnesota Minneapolis Minnesota USA; ^17^ Division of Kidney Diseases and Hypertension Northwell Health, Zucker School of Medicine at Hofstra/Northwell Great Neck New York USA; ^18^ University Hospital La Fe, Universidad Catolica “San Vicente Martir”, Instituto de Investigation Sanitaria La Fe Valencia Spain

**Keywords:** acute renal failure, cast nephropathy, multiple myeloma

## Abstract

Acute renal failure due to cast nephropathy (CAN) is a severe complication of multiple myeloma (MM). Here, we aimed to identify parameters associated with renal outcomes and survival in newly diagnosed MM patients with CAN‐related acute renal failure and to validate the IMWG criteria for renal response. CAN diagnosis was based on biopsy or clinical assessment. Of 787 registered patients, 354 met the inclusion criteria, requiring at least two therapy cycles with documented chemotherapy response, renal response, and eGFR. Baseline free light chain (FLC) levels (median 6825.65 mg/L) decreased below 500 mg/L in 67.8% of patients. According to IMWG classification, renal complete response, partial response, and minimal response were achieved in 33.9%, 19.5%, and 28.0% of patients, respectively. The best eGFR values > 60 mL/min/1.73 m^2^ were observed in 33.9% of patients, while 33.3%, 22.3%, and 10.5% had best eGFR values of 30–59, 15–29, and < 15 mL/min/1.73 m^2^, respectively. Renal response correlated with baseline FLC levels, IgG kappa, eGFR, and ECOG status in multivariate analysis, as well as with myeloma response and FLC reduction in univariate analysis. Median overall survival was 77.6 months. Survival correlated with age, myeloma response, ECOG status, FLC kappa type, baseline eGFR, standard‐risk cytogenetics, and calcium levels, among other factors. A comparison of IMWG renal response criteria with classification based solely on best eGFR showed similar utility. Of 136 patients requiring dialysis, 80 (58.8%) were able to discontinue dialysis during therapy. Bortezomib containing myeloma therapy, along with significant FLC reduction, was associated with favorable outcome.

## Graphical abstract

Disease‐specific, patient‐specific, and treatment‐related factors correlate with renal recovery.

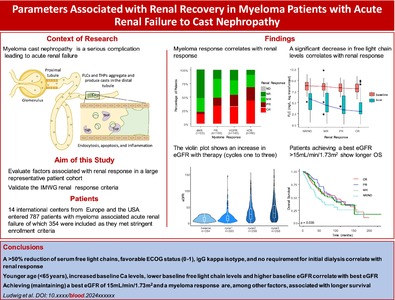

## 1 Introduction

Acute renal failure due to cast nephropathy is one of the major complications of multiple myeloma. Previous statistics suggest an incidence of 12%–17% of stage IV and stage V renal failure (eGFR < 30 mL/min) among newly diagnosed patients [1] but with earlier diagnosis and therapeutic intervention in multiple myeloma, these figures may be lower nowadays. Cast nephropathy (CAN) is the most common form of monoclonal gammopathy‐related acute kidney damage [2], accounting for about 60% of monoclonal protein–induced renal pathologies [3] and requires intervention to rescue kidneys from irreversible damage [4, 5]. CAN results from the aggregation and precipitation of filtered monoclonal light chains with Tamm‐Horsfall protein in the distal tubules [6]. These casts obstruct and damage distal renal tubules and trigger a cellular reaction, including macrophage‐derived giant cells [7], and promote inflammatory cytokine secretion leading to interstitial fibrosis [8]. This process is worsened by low urine pH, high calcium/phosphate levels, dehydration, use of loop diuretics, NSAIDs, and infection or inflammation [9, 10] and may result in irreversible tubular atrophy and interstitial fibrosis [11]. The variability in free light chain (FLC) toxicity is generally attributed to the CDR3 (third complementarity‐determining region) of the variable domain of kappa or lambda light chain molecules, which determines the binding avidity to a nine‐amino‐acid sequence on Tamm‐Horsfall protein [6].

Restoration of renal function depends on the degree of interstitial fibrosis and the number of casts at the start of treatment intervention [12, 13]. Provision of supportive care, such as hydration, hypercalcemia correction, and avoidance of nephrotoxins, and most importantly, reduction of excessive light chain production [4] through prompt diagnosis and initiation of effective anti‐myeloma therapy [14, 15] is of utmost importance. Renal improvement is assessed by using the criteria defined by the International Myeloma Working Group (IMWG) [16], based on eGFR changes.

Previous studies aiming to identify parameters indicative of renal recovery and response to myeloma therapy have often been limited by small patient cohorts [17, 18]. Here, we present an international collaboration among 14 international centers specializing in MM and/or renal diseases. The primary goal of this cooperation was to assess the impact of patient characteristics, disease features, and treatment‐related factors on renal outcomes and overall survival, while also validating the IMWG criteria for renal response in a large cohort of patients with CAN and newly diagnosed multiple myeloma.

## 2 Patients and Methods

The project was discussed during the regular meetings of the IKMG (International Kidney and Monoclonal Gammopathy Group), held in conjunction with the ASH meetings. The IKMG brings together the world experts in kidney disease caused by monoclonal proteins. During these discussions, it was decided to invite 35 centers to participate. Ultimately, 14 of these centers provided patient‐level data for 787 patients, which were entered in the specially built study database hosted by REDCap (Table S1). Of these, 399 patients presented with an eGFR of < 40 mL/min/1.73 m^2^, and 354 patients had renal disease stage IV (eGFR 15–29 mL/min/1.73 m^2^) or stage V (eGFR < 15 mL/min/1.73 m^2^).

Since outcome differences were similar between the two groups, although differences were more pronounced in the latter cohort, we present all analysis conducted in patients with stage IV and V renal failure. The clinical diagnosis of cast nephropathy was based on the presence of acute renal failure (GFR < 30 mL/min), multiple myeloma, elevated serum free light chains (> 500 mg/L), and proteinuria. The updated IMWG criteria suggest that serum free light chain levels above 500 mg/mL in the presence of myeloma and acute renal failure are highly indicative of cast nephropathy [19]. Patients presenting with anuria or serum free light chain concentrations below 500 mg/L should undergo renal biopsy to confirm the diagnosis; this procedure was performed in 119 patients. An additional 235 patients were diagnosed based on clinical criteria, as recommended by the IMWG [11, 19]. Survival analysis of both groups showed similar survival (Figure S1). Further inclusion criteria required documentation of at least two therapy cycles, eGFR calculation using the Modification of Diet in Renal Disease (MDRD) formula [20], and best response to myeloma therapy (Figure S2). IMWG defined renal response criteria, and stages of kidney disease and myeloma response criteria are shown in Tables 1 and S2.

**TABLE 1:** Renal response criteria as defined by the IMWG.

Renal response	Baseline eGFR (mL/min/1.73 m^2^)	Best eGFR (mL/min/1.73 m^2^)
Complete response	< 50	≤ 60
Partial response	< 15	30–59
Minor response	15–29	30–59
Minor response	< 15	15–29
Non‐definable	15–29	30–59

### 2.1 Statistical Analysis

Statistical analyses were performed using SPSS software version 17.0 (IBM SPSS Statistics, IBM Corporation, Armonk, NY) and RStudio (version 4.3.3; R Foundation for Statistical Computing, Vienna, Austria). Continuous variables are presented as medians (range) and categorical variables as counts (percentages). Kaplan‐Meier [21], Cox–, binary logistic—and generalized linear regression analyses were used to assess survival and renal response. For the regression analyses, variables were transformed as needed. To identify the most parsimonious and predictive model, a bidirectional stepwise approach based on the Akaike Information Criterion (AIC) was used. Since a prerequisite for this procedure is a complete dataset, missing data were imputed via predictive mean matching to avoid the drawbacks of simple imputation methods such as median substitution. A single imputed dataset was created to ensure compatibility with the model selection procedures, which are not natively supported by multiple imputed datasets. Receiver operating characteristics (ROC) curves compared to the IMWG criteria versus best eGFR. Landmark analysis at 3 and 10 months for myeloma response and renal response was included. The cutoff times were chosen based on median times to best response.

## 3 Results

### 3.1 Baseline Patient Characteristics

Baseline characteristics are shown in Table 2. Patients were diagnosed between 1999 and 2022.

**TABLE 2:** Patient characteristics.

Parameter	*n*	Percent/median (range)
Demographic characteristics		
Age (median, range)	351	62.5 (35–93)
Gender (female/male)	167/187	40.8%/59.2%
Myeloma characteristics		
IgG/IgA/IgD/IgM/LC only	139/47/8/2/131	39.3%/13.3%/2.3%/0.6%/37%
*κ* LC, mg/L (median, range)	203	4790 (8.9–201 000)
*λ* LC, mg/L (median, range)	144	3585 (57.3–51 000)
FLC only, mg/L (median, range)	131	6825.65 (379–201 000)
*κ* LC only, mg/L (median, range)	81	7018.65 (379–201 000)
*λ* LC only, mg/L (median, range)	50	5645 (534–51 000)
FLC of IgG/IgA/IgD/IgM, mg/L (median, range)^a^	196	3390 (8.9–49 100)
Bone marrow plasma cells % (median, range)	274	60 (4–100)
Hemoglobin, g/L (median, range)	353	9.2 (4.4–15.7)
LDH, U/L (median, range)	280	217.5 (53–1100)
*ß*2 microglobulin mg/L (median, range)	323	16 (1.8–85.9)
ISS stage I + II/III	43/304	12.1%/85.9%
ECOG‐status 0/1/2/3/4	56/170/79/31/6	15.3%/48.0%/22.3%/8.8%/1.7%
Hypercalcemia 0/1/2/3/4	220/34/19/19/30	62.1%/9.6%/5.4%/5.4%/8.5%
HRCA 0/1/ ≥ 2	113/79/39	39.1%/22.3%/11.0%
Renal characteristics		
Diagnosis by clinical parameters	272	76.8%
Diagnosis by renal biopsy	81	22.9%
eGFR, < 15 mL/min/173 m^2^	209	59.0%
eGFR, 15–29 mL/min/173 m^2^	145	41.0%
Proteinuria, g/day (median, range)	226	2042 (0–38 000)
Dialysis	136	38.4%
Continued dialysis	56	41.2%

*Note*: Missing data: age 3 (0.9%), type of Myeloma 27 (7.6%), FLC concentration 5 (1.4%), FLC type 6 (1.7%), Hemoglobin 1 (0.003%), LDH 74 (20.9%), *ß*2 microglobulin 31 (8.8%), ISS stage 7 (2.0%), ECOG‐Status 12 (3.4%), Hypercalcemia 32 (9.0%), HRCA 123 (34.8%), diagnosis 1 (0.003%), dialysis 2 (0.6%), continued dialysis 8 (2.3%), Proteinuria 128 (36.2%).

^a^ Includes patients with intact immunoglobulin isotype plus free light chains.

Baseline eGFR was < 15 mL/min/1.73 m^2^ in 209 patients (59%) and between 15 and 29 mL/min/1.73 m^2^ in 145 patients (41%), with a median eGFR of 12.32 mL/min/1.73 m^2^. One hundred thirty‐six (38.4%) patients required dialysis at diagnosis. Median baseline proteinuria was 2042 mg/24 h. Median free light chain levels were 4790 mg/L (kappa) and 3585 mg/L (lambda); 31% had hypercalcemia. The median time to therapy was 9 days, with a median follow‐up of 35.9 months.

### 3.2 Myeloma Therapy

Treatment initiation within 5 days after diagnosis was associated with longer survival (median 90 vs. 51 months), although the difference did not reach levels of statistical significance (*p* = 0.095). Myeloma therapy mainly involved bortezomib‐based regimens, with 34.8% receiving CyBorD/VCD and 43.4% other bortezomib‐based combinations, resulting in nearly 80% of patients receiving bortezomib as part of their first‐line therapy (Table 3). Few patients received daratumumab, and none received bispecific antibodies.

**TABLE 3:** Myeloma therapy and myeloma and renal response.

Parameter	*n*	Value
Duration of follow up (median)	354	39.3 months
First line chemotherapy		
Cybord (VCD)	111	34.8%
Vd	38	11.%
VTd	23	7.2%
PAD	20	6.3%
VRd	18	5.6%
Rd	11	3.4%
VDTC	10	3.1%
VMDT	10	3.1%
VDRC	10	3.1%
Other bortezomib containing regimens	12	3.7%
Other regimens without bortezomib	56	17.6%
Myeloma response		
CR including MRDneg	87	24.3%
VGPR	145	41.0%
PR	100	28.2%
MR	9	2.5%
SD	8	2.3%
PD	6	1.7%
Light chain response		
Lowest FLC achieved during treatment (median, range)	334	42.05 (0.06–34 700)
Kappa (median, range)	203	40.68 (0.06–24 800)
Lambda (median, range)	144	46.6 (0.06–34 700)
FLC < 500 mg/L	334	67.8%
> 50% reduction in FLC at cycle 2 or 3	252	58.5%
Lowest FLC achieved in patients with ≥ VGPR (median, range)	334	21.4 (0.06–15 600)
Renal response (IMWG)		
CR	120	33.9%
PR	69	19.5%
MR	99	28.0%
ND^a^	2	0.6%
Renal response (Best eGFR, mL/min/1.73 m^2^)		
≥ 60	120	33.9%
30–60	118	33.3%
15–29	79	22.3%
< 15	37	10.5%
Dialysis		
Continued dialysis until death/end of FU	56	15.8%
Dialysis discontinued	136	38.4%

*Note*: Missing data: chemotherapy 35 (9.9%), Lowest FLC concentration 20 (5.7%), FLC type 6 (1.7%), FLC reduction 102 (28.8%), dialysis 2 (0.6%), continued dialysis 8 (2.3%).

^a^ Non‐definable according to IMWG criteria (Patients with eGFR 15– < 30, with an increase in eGFR to ≥ 30 – < 40).

### 3.3 Myeloma and Renal Response

**FIGURE 1:**
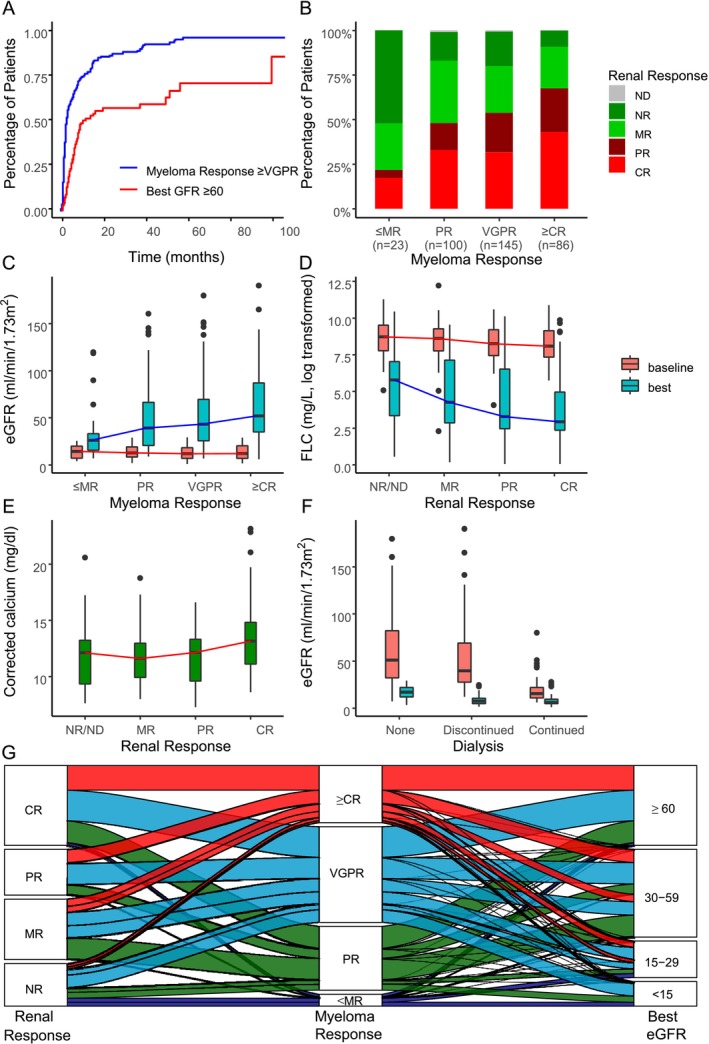
(A) Cumulative incidence of myeloma and renal (best eGFR) response. (B) Renal response (IMWG) by myeloma response. (C) best eGFR by myeloma response. (D) baseline and best eGFR by renal response category (IMWG). (E) Calcium levels by renal response (IMWG) category. (F) Baseline and best eGFR by dialysis status. (G) Sankey diagram correlating myeloma response with renal response (IMWG) and best eGFR. [Color figure can be viewed at wileyonlinelibrary.com]

A myeloma response (best response) was achieved in 93.5% (24.3% ≥ CR/MRD negativity, 41.0% VGPR, 28.2% PR); major renal responses (best response CRrenal or PRrenal) were achieved in 189 patients (33.9% CRrenal, 19.5% PRrenal) and MRrenal in 28.0%. Myeloma response (≥ VGPR) correlated with renal response (≥ PRrenal) (OR 1.89, *p* = 0.005; Figure 1B). Free light chain reductions to < 500 mg/L were achieved in 67.8% and > 50% reduction of FLC by cycles 2 or 3 in 58.5% of patients (Table 3). Best eGFR improvements were seen in 33.9% (> 60 mL/min/1.73 m^2^), 33.3% (30–59), 22.3% (15–29), with 10.5% remaining < 15 mL/min/1.73 m^2^. An overall increase in eGFR with progressing treatment cycles was observed (Figure S3). Median times to best myeloma and eGFR responses were 2.46 and 10.1 months, respectively, with further improvements beyond 3 years in 11% and 43.1% of patients (Figure 1A). Landmark analysis based on the median times to best response showed a continuation of the survival benefit of a myeloma response, whereas the survival differences across renal response groups decreased (Figure S4).

### 3.4 Regression Analysis for Factors Correlating With Renal and Myeloma Response

In regression analyses, renal response correlated in multivariate models with ECOG status (OR 0.61, *p* = 0.002), IgG kappa isotype (OR 2.72, *p* = 0.018), dialysis (OR 0.53, *p* = 0.034), and > 50% FLC reduction (OR 2.59, *p* = 0.003); best eGFR was associated with age (Coeff −0.62, *p* < 0.001), calcium (Coeff 3.05, *p* < 0.001), baseline FLC (Coeff −4.86, *p* < 0.001), baseline eGFR (Coeff 0.80, *p* = 0.005), dialysis (Coeff −10.80, *p* = 0.001), and > 50% FLC reduction (Coeff 9.24, *p* = 0.021). In univariate regression, myeloma response correlated with best eGFR (Coeff 8.53, *p* = 0.034; Figure 1C). Baseline free light chain levels correlated with both best eGFR (Coeff −72.99, *p* = 0.006; Figure 1D) and renal response (OR 0.79, *p* = 0.047), with similar results for *λ* and *κ* types; calcium correlated with best eGFR (Coeff 4.41, *p* < 0.001) but showed a non‐significant tendency with better renal response (OR 1.09, *p* = 0.138) (Table 4, Figure 1E).

**TABLE 4:** Simple generalized linear regression of clinical or laboratory data with best eGFR, and binary logistic regression analysis of clinical or laboratory data with ≥ MRrenal.

Clinical data	Simple generalized linear regression with best GFR					Binary logistic regression with ≥ MRrenal				
	*N*	Univariate		Multivariate		*N*	Univariate		Multivariate	
Variable		Coefficient (95% CI)	*p*	Coefficient (95% CI)	*p*		OR (95% CI)	*p*	OR (95% CI)	*p*
Patient and disease characteristics										
Age	348	−0.54 (−0.85–(−0.23))	< 0.001***	−0.62 (−0.91–(−0.34))	< 0.001***	351	0.99 (0.96–1.00)	0.397		
ISS stage III	343	−11.74 (−23.33–(−0.15))	0.048*			347	0.55 (0.18–1.33)	0.224		
LDH	278	−170.79^a^ (−1360.13–1018.55)	0.779			280	0.79^a^ (0.09–8.68)	0.833		
*ß*2 microglobulin	319	−0.53 (−0.84–(−0.22))	< 0.001***			323	0.98 (0.98–1.01)	0.038*		
Hemoglobin	349	2.56 (0.55–4.56)	0.013*			353	1.11 (0.96–1.30)	0.169		
Calcium (corrected)	315	4.41 (2.99–5.83)	< 0.001***	3.05 (1.70–4.39)	< 0.001***	322	1.09 (0.98–1.23)	0.138	1.10 (0.98–1.25)	0.118
ECOG‐status	338	0.21 (−3.99–4.41)	0.922			340	0.70 (0.52–0.93)	0.014*	0.61 (0.45–0.83)	0.002**
M‐protein										
FLC type (kappa vs. Lambda)	344	7.86 (0.17–15.55)	0.046*	5.15 (−1.94–12.24)	0.155	348	1.37 (0.80–2.36)	0.253		
IgG kappa Isotype	323	12.18 (2.67–21.70)	0.013*	6.71 (−1.81–15.23)	0.124	327	2.04 (0.97–4.85)	0.079	2.72 (1.25–6.68)	0.018*
FLC levels	345	−72.99^b^ (−125.02–(−20.95))	0.006**	−4.86^b^ (−7.69–(−2.03))	< 0.001***	349	0.79^b^ (0.63–0.99)	0.047*		
FLC kappa levels	202	−67.50^b^ (−128.72–(−6.28))	0.032*			203	0.89^b^ (0.65–1.20)	0.443		
FLC lambda levels	141	−43.90^b^ (−75.31–(−12.49))	0.007**			144	0.64^b^ (0.43–0.91)	0.016**		
Proteinuria	224	−9.70^a^ (−45.38–25.99)	0.595			226	0.51^a^ (0.06–5.89)	0.547		
Renal function										
GFR (baseline)	350	1.42 (0.93–1.91)	< 0.001***	0.80 (0.25–1.34)	0.005**	354	1.02 (0.99–1.06)	0.203		
Diagnosis by biopsy	349	−12.26 (−21.21–(−3.32))	0.008**			353	0.83 (0.45–1.57)	0.547		
Dialysis	348	−20.51 (−28.01–(−13.02))	< 0.001***	−10.80 (−19.23–(−2.39))	0.001**	352	0.52 (0.30–0.90)	0.018*	0.53 (0.30–0.96)	0.034*
Cytogenetics										
HRCA (intermediate risk, ≥ 1)	229	−6.67 (−16.40–3.07)	0.181			231	0.48 (0.23–0.99)	0.050		
HRCA (high risk, ≥ 2)	229	−1.02 (−11.98–14.01)	0.878			231	0.59 (0.26–1.44)	0.225		
Treatment response										
Myeloma response (≥ VGPR)	350	8.53 (0.67–16.39)	0.034*			354	1.62 (1.22–2.95)	0.084		
FLC reduction > 50% at cycle 2 or 3	269	18.75 (7.59–29.90)	0.001**	9.24 (1.44–17.03)	0.021*	252	3.44 (1.73–6.78)	< 0.001***	2.59 (1.37–4.82)	0.003**
FLC reduction > 90%	269	16.25 (6.93–25.57)	< 0.001***			252	2.79 (1.51–5.17)	0.001**		
Bortezomib	348	−2.34 (−14.62–9.94)	0.709			352	0.82 (0.29–1.93)	0.677		

^a^ Unit change of 1%.

^b^ Log‐transformed. Bold values inidcate statistically significant results (*p <* 0.05). * *p* < 0.05** *p <* 0.01*** *p <* 0.001.

### 3.5 Overall Survival

**FIGURE 2:**
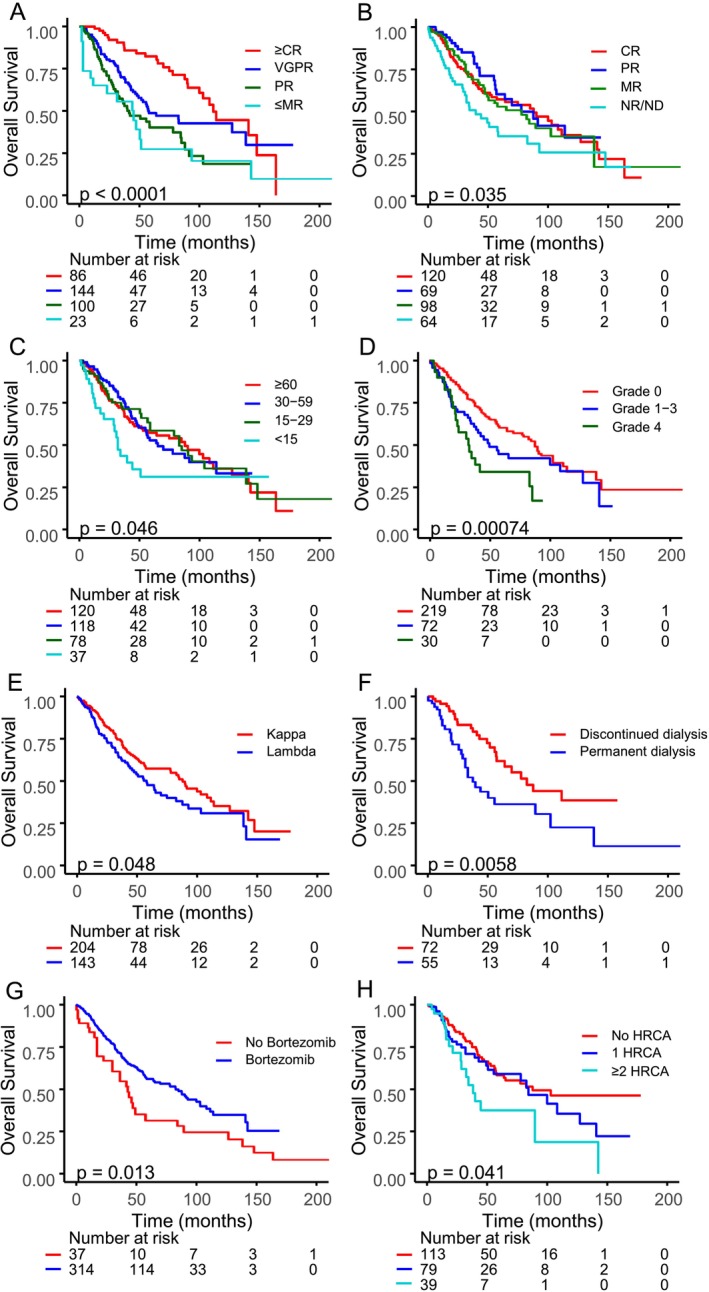
(A) Overall survival by myeloma response. (B) Overall survival by renal response. (C) Overall survival by eGFR category. (D) Overall survival by calcium serum levels. (E) Overall survival by free light chain type. (F) Overall survival by dialysis continuation/discontinuation. (G) Overall survival by bortezomib‐containing regimen. (H) Overall survival by number of cytogenetic aberrations. [Color figure can be viewed at wileyonlinelibrary.com]

Of the 787 patients included in our database, 58 (7.4%) died before day 61 and were not included in this analysis. Median overall survival of the study population of 354 patients was 77.6 months. Favorable factors included ≥ VGPR (99.8 vs. 42.0 months; HR 0.49, *p* < 0.001), > 50% FLC reduction (77.6 vs. 37.4 months; HR 0.58, *p* = 0.009), best eGFR ≥ 15 mL/min/1.73 m^2^ (82.6 vs. 31.9 months; HR 0.52, *p* = 0.006), bortezomib‐based therapy (82.6 vs. 43.5 months; HR 0.59, *p* = 0.014), and *κ* light chain type (87.8 vs. 57.8 months; HR 0.60, *p* = 0.002) (Table 5, Figure 2A,E,G). Shorter survival was linked to hemoglobin ≤ 12 g/dL (HR 2.82, *p* = 0.004), hypercalcemia (HR 1.70, *p* = 0.002), ISS stage III (58.4 vs. 104.7 months; HR 3.25, *p* < 0.001), ECOG III–IV (28.5 vs. 90.2 months; HR 1.73, *p* < 0.001), older age (HR 1.05, *p* < 0.001), elevated *λ* FLC (HR 4.85, *p* = 0.015), ≥ 2 high‐risk cytogenetic aberrations (87.8, 84.0, and 38.2 months for 0, 1, and ≥ 2 aberrations; HR 1.85, *p* = 0.020), high LDH (HR 1.85, *p* < 0.001), and elevated β2‐microglobulin (HR 1.02, *p* < 0.001) (Table 5, Figure 2D.H). Patients with ≥MR renal had longer OS (84.3 vs. 44.0 months; HR 0.63, *p* = 0.015) (Table 5, Figure 2B), as did those with eGFR ≥ 15 mL/min/1.73 m^2^ (82.6 vs. 31.9 months; HR 0.52, *p* = 0.006) (Table 5, Figure 2C). OS varied by renal response (CR 87.8, PR 77.6, MR 77.9, NR 39.6 months; *p* = 0.035) and best eGFR (< 15: 31.9, 15–< 30: 82.6, 30–< 60: 65.2, ≥ 60: 87.8 months; *p* = 0.046) (Table 5, Figure 2B,C).

**TABLE 5:** Cox regression analysis with overall survival.

Clinical data		Univariate		Multivariate	
Variable	*N*	HR (95% CI)	*p*	HR (95% CI)	*p*
Patient and disease characteristics					
Age	350	1.05 (1.04–1.07)	< 0.001***	1.06 (1.04–1.08)	< 0.001***
ISS stage III	346	3.25 (1.65–6.37)	< 0.001***		
LDH ≥ 240	280	1.85 (1.30–2.64)	< 0.001***	2.16 (1.53–3.03)	< 0.001***
*ß*2 microglobulin	322	1.02 (1.01–1.03)	< 0.001***	1.02 (1.00–1.03)	0.007**
Hemoglobin ≤ 12	352	2.82 (1.38–5.77)	0.004**	1.65 (0.79–3.46)	0.184
Hypercalcemia	321	1.70 (1.22–2.38)	0.002**	1.73 (1.24–2.41)	0.003**
Bone marrow plasma cells	274	1.01 (1.00–1.01)	0.013*		
ECOG‐status	341	1.73 (1.56–1.91)	< 0.001***	1.44 (1.20–1.73)	< 0.001***
M‐protein					
FLC type (kappa vs. lambda)	348	0.73 (0.53–1.00)	0.053	0.60 (0.44–0.83)	0.002**
IgG kappa Isotype	326	0.89 (0.61–1.30)	0.561		
FLC levels	348	0.67 (0.09–4.85)^a^	0.692		
FLC kappa levels	203	0.31 (0.02–4.95)^a^	0.410		
FLC lambda levels	143	4.85 (1.36–17.31)^a^	0.015*		
Proteinuria	226	1.81 (0.36–9.20)^a^	0.474		
Renal function					
Baseline GFR	354	1.00 (0.98–1.02)	0.860	1.04 (1.01–1.07)	0.003**
Diagnosis by Biopsy	352	1.15 (0.80–1.67)	0.438		
Dialysis	351	1.08 (0.78–1.48)	0.656	1.61 (1.04–2.49)	0.031*
Cytogenetics					
HRCA (intermediate risk, ≥ 1)	231	1.44 (0.96–2.17)	0.080		
HRCA (high risk, ≥ 2)	231	1.85 (1.10–3.13)	0.020*	1.65 (1.14–2.40)	0.009*
Myeloma response					
Myeloma response (≥ VGPR)	353	0.49 (0.36–0.68)	< 0.001***	0.47 (0.34–0.65)	< 0.001***
FLC reduction > 50% at cycle 2 or 3	272	0.58 (0.39–0.88)	0.009**		
FLC reduction > 90%	272	0.71 (0.80–1.02)	0.065		
Bortezomib	351	0.59 (0.39–0.90)	0.014*		
Renal response					
CRrenal	353	0.93 (0.67–1.30)	0.687		
≥ PRrenal	353	0.78 (0.57–1.07)	0.124		
≥ MRrenal	353	0.63 (0.43–0.92)	0.015*		
Best GFR	349	0.46 (0.20–1.06)^a^	0.068		
Best GFR ≥ 60 (same as CRenal)	353	0.93 (0.67–1.30)	0.687		
Best GFR ≥ 30	353	0.81 (0.58–1.13)	0.216		
Best GFR ≥ 15	353	0.52 (0.33–0.83)	0.006**		

^a^ Unit change of 1%. Bold values inidcate statistically significant results (*p <* 0.05). * *p <* 0.05** *p <* 0.01*** *p <* 0.001.

In multivariate analysis, independent predictors of survival were age (HR 1.06, *p* < 0.001), LDH ≥ 240 U/L (HR 2.16, *p* < 0.001), *β*2‐microglobulin (HR 1.02, *p* = 0.007), hypercalcemia (HR 1.73, *p* = 0.003), ECOG status (HR 1.44, *p* < 0.001), *κ* FLC type (HR 0.60, *p* = 0.002), baseline eGFR (HR 1.04, *p* = 0.003), dialysis requirement (HR 1.61, *p* = 0.031), presence of ≥ 2 high risk cytogenetic aberrations (HR 1.65, *p* = 0.009), and myeloma response (HR 0.47, *p* < 0.001) (Table 5).

### 3.6 Validation of IMWG Renal Response Criteria With eGFR Categories

Comparing the IMWG renal response criteria with categories of best eGFR, the IMWG criteria had a slightly smaller C‐index (0.68 vs. 0.70) but a marginally better predictive power in Cox regression (*R* ^2^: 0.031 vs. 0.019). When used as a single predictor for survival, the IMWG criteria had a slightly higher ROC survival prediction at 1 year (AUC: 0.626 vs. 0.595) compared to best eGFR criteria. Similar results were obtained in landmark analyses (Table S3).

### 3.7 Patients With Renal Biopsy

Among 81 (22.9%) patients undergoing both renal biopsy and clinical evaluation, baseline eGFR was lower (10.6 vs. 13.02 mL/min/1.73 m^2^; *p* = 0.005), and best eGFR was reduced (36.0 vs. 54.39 mL/min/1.73 m^2^; *p* = 0.013) compared to those assessed clinically, though survival was similar (77.9 vs. 74.7 months; *p* = 0.413) (Figure S1).

### 3.8 Patients on Dialysis

Of 136 (38.4%) patients initially requiring dialysis, 56 (41.2%) remained on dialysis while 80 (58.8%) discontinued it; those who stopped dialysis had higher best median eGFR (39.5 vs. 15.3 mL/min/1.73 m^2^; *p* < 0.001) (Figure 1F) and longer OS (82.6 vs. 31.9 months; *p* = 0.005) (Figure 2F). Overall survival was notably longer in patients achieving a best eGFR of 15 mL/min/1.73 m^2^ or higher (82.6 months) compared to those with lower eGFR levels (31.9 months; *p* = 0.005). None of the patients received dialysis with cut‐off membranes [22, 23].

## 4 Discussion

This study represents the largest cohort of patients with cast nephropathy, comprising data from 14 international myeloma and renal study groups. Focusing on patients with an estimated glomerular filtration rate (eGFR) of less than 30 mL/min/1.73 m^2^, stage IV and V renal failure, we observed an International Myeloma Working Group (IMWG)‐defined renal response rate of 53.4%, including 33.9% complete renal responses (CRrenal) and 19.5% partial renal responses (PRrenal). When using eGFR as a response criterion, 33.3% of patients improved to an eGFR of 30–59 mL/min/1.73 m^2^, and 33.9% achieved ≥ 60 mL/min/1.73 m^2^, representing an overall improvement of 67.2%. The strong concordance between IMWG criteria and eGFR‐based assessments was evident, as both methods yielded similar survival curves, emphasizing the validity of the IMWG criteria for renal response, which take baseline eGFR levels into account. This agreement was also supported by receiver operating curve analysis and the Sankey diagram [24], which showed a similar distribution of the renal response criteria in patients with different myeloma responses (Figure 1G).

Our study identified several factors associated with renal recovery and overall survival. The median time to achieve a myeloma response greater than very good partial response (VGPR) was 2.46 months, while the median time to best eGFR (≥ 60) was 10.1 months. This is consistent with a previous report indicating a maximum cumulative incidence rate for CRrenal of 46% in patients with baseline eGFR < 50 mL/min/1.73 m^2^ one year after initiation of therapy [25]. The extended follow‐up in our study showed that myeloma response and eGFR continued to improve in 11% and 43.1% of patients, respectively, over 3 years. This may primarily be attributed to the use of effective new therapies after relapses to first‐line treatment and the longer follow‐up period.

The most important predictors of renal recovery according to IMWG criteria included baseline Eastern Cooperative Oncology Group (ECOG) status (0–2 vs. 3–4), IgG kappa isotype, absence of dialysis, and reduction of free light chains (FLC) by more than 50% at cycle 2 or 3. Both FLC reduction and kappa FLC isotype have previously been identified as significant predictors of renal recovery [4]. Early intervention is critical, as reabsorption of FLCs in the proximal tubule can induce a proinflammatory state that promotes cast formation, apoptosis, and obstruction of the distal tubules and possible irreversible renal damage [26].

When evaluating factors associated with best eGFR values, significant correlations were observed with age, baseline FLC levels, eGFR, calcium levels, dialysis status, and FLC reduction of more than 50%. Hypercalcemia, which was present in 31% of our patients at baseline, is a hallmark of progressive myeloma and is known to impair renal function by decreasing the sensitivity of collecting ducts to antidiuretic hormone, reducing sodium reabsorption, increasing diuresis, and constricting afferent arterioles. Immediate correction of hypercalcemia by hydration and bisphosphonate therapy can increase the likelihood of renal recovery, as previously reported by other groups [27, 28, 29].

Higher baseline eGFR levels indicate less severe renal damage, offering a greater chance for tissue repair and functional improvement. In particular, kappa light chains exhibit a lower tendency to bind Tamm‐Horsfall protein and form large obstructive casts [30] contributing to better outcomes in patients with IgG kappa paraprotein. Conversely, lambda light chains are less soluble, making them more susceptible to aggregation, dimer formation, hyaline cast deposition, and tubular damage, increasing the risk of acute kidney injury [31].

Early treatment initiation within 5 days after diagnosis was associated with a tendency for improved survival. The median overall survival (OS) for the entire cohort was 77.6 months, and correlated with factors such as age, lactate dehydrogenase (LDH), β2‐microglobulin, hemoglobin, and ECOG status. Multivariate analysis revealed strong associations between OS and myeloma response category, FLC type, baseline eGFR, calcium levels, and the presence of two or more high‐risk cytogenetic aberrations.

Consistent with observations from other groups [32, 33], bortezomib‐based combinations were associated with improved OS in univariate analysis. Bortezomib efficacy may be attributed to its inhibition of nuclear factor‐kappa B (NF‐*κ*B), which is upregulated by intracellular uptake of light chains. Bortezomib suppresses the release of inflammatory cytokines and promotes anti‐apoptotic gene expression in epithelial cells of the proximal tubules [34]. Both activities may explain the high activity of this key anti‐myeloma drug in cast nephropathy [34].

Patients who achieved a renal response, defined either by IMWG criteria (≥MRrenal) or an eGFR > 15 mL/min/1.73 m^2^, had a significantly longer OS. It is noteworthy that OS was shorter in patients with involved lambda light chains compared to those with kappa light chains, which is consistent with previous reports [35, 36]. A reduction of FLCs of more than 50% correlated with both renal response and best eGFR. Previous studies have associated overall survival with different levels of achieved eGFR [13, 32, 35], while more recent studies support our findings that there are only marginal differences in survival between patients with an eGFR of ≥ 15 mL/min/1.73 m^2^ and renal recovery to higher eGFR categories [36, 37, 38].

Of 38% of patients who initially required dialysis, 58.8% were able to discontinue dialysis. This figure is consistent with findings of the EuLITE study [23] and slightly lower than in the MYRE study [22], both of which compared high cut‐off dialysis membranes with conventional hemodialysis. Patients who discontinued dialysis had a significantly higher median best eGFR (39.5 vs. 15.3 mL/min/1.73 m^2^) and longer survival (82.4 vs. 38.2 months) than patients who remained on continuous dialysis. These findings align with other studies reporting variable percentages of dialysis discontinuation [39].

Limitations of this study include its retrospective design, the exclusion of patients dying before the end of cycle 2, missing data points, and the heterogeneity of treatment regimens used for first‐line therapy. Only about 20% of patients underwent renal biopsy. It is recognized that a detailed analysis of biopsies could provide valuable information, such as the number of casts, the degree of interstitial fibrosis and tubular atrophy, and the prevalence of pathologies other than cast nephropathy [13, 40]. Previous data suggest that the combination of the diagnosis of multiple myeloma, with light chain proteinuria and serum FLC levels > 500 mg/L is highly indicative of cast nephropathy [11, 16]. The likelihood of tubular casts formation increases with rising FLC levels, particularly when the threshold of 500 mg/mL is exceeded [23]. Notably, overall survival was very similar between patients who underwent renal biopsy compared with those who did not (Figure S1). Another limitation is the bidirectional stepwise procedure that was applied for the variable selection for the multivariate models as it can lead to overfitting and unstable results in small samples. Nevertheless, it is still preferred to the disadvantages entailed by including variables that show statistical significance in univariate regression [41].

The median follow‐up time of our study was more than 3 years, which partly explains why daratumumab, which has been approved for first‐line therapy only recently, was not used in our patient cohort. Recent studies employing daratumumab with Vd (bortezomib and dexamethasone) or VCD (bortezomib, cyclophosphamide, and dexamethasone) demonstrated rapid and substantial FLC reduction with high rates of myeloma and renal responses [42, 43, 44]. The time to achieve an FLC reduction of greater than 50% was much shorter compared with conventional chemotherapy, with one study reporting a median of only 3 days [42]. Given these recent excellent results, CD38 antibody combinations with Vd or VCd should strongly be considered in any patient with acute renal failure due to cast nephropathy. Whether bispecific or trispecific antibodies will achieve similar results remains to be demonstrated. Preliminary reports with teclistamab [45] and elranatamab [46] in patients with end‐stage renal failure suggest that myeloma response is not impaired by advanced renal failure, and the potential for renal recovery remains. CAR‐T cells have been used in patients with renal failure [47], but the logistic requirements preclude their use in patients with acute renal failure, where timely treatment initiation is mandatory. Strengths of this study include the international collaboration of 14 study groups, the large dataset ensuring robust results, and the conclusive validation of the IMWG response criteria.

In conclusion, our study highlights the importance of effective myeloma treatment, including bortezomib‐based regimens, substantial FLC reduction, and management of hypercalcemia for improving renal outcomes and survival in patients with acute renal failure due to cast nephropathy. Other predictors of long overall survival were normal LDH, low *ß*2‐microglobulin levels, kappa FLC type, higher baseline eGFR (> 15 mL/min/1.73 m^2^), absence of cytogenetic high‐risk factors, and no dialysis requirement at diagnosis.

## Ethics Statement

The study was approved by the Ethics Committee of the Viennese Healthcare Group (EK‐20‐101‐VK), as well as by the respective ethics boards of all participating centers.

## Conflicts of Interest

H.L.: Consulting and/or speakers bureau for AMGEN, Takeda, Celgene‐BMS, Janssen, Pfizer, Sanofi; Research funding: AMGEN and Sanofi; M.A.D.: Consultancy for Amgen, Sanofi, Regeneron, Menarini, Takeda, GSK, BMS, Janssen, Beigene, Swixx, Astra Zeneca; Honoraria from: Amgen, Sanofi, Regeneron, Menarini, Takeda, GSK, BMS, Janssen, Beigene, Swixx, Astra Zeneca; Speaker's Bureau or Advisory Committees for Amgen, Sanofi, Regeneron, Menarini, Takeda, GSK, BMS, Janssen, Beigene, Swixx and AstraZeneca; E.T.: Advisory Boards for Amgen, AstraZeneca, EUSA Pharma, BMS, GSK, J&J, Menarini/Stemline, Pfizer, Sanofi, Takeda; Honoraria from Amgen, Antengene, AstraZeneca, EUSA Pharma, BMS, Forus, GSK, J&J, Menarini/Stemline, Novartis, Pfizer, Sanofi, Swixx, Takeda; Research Funding from Amgen, GSK, J&J, Sanofi, Takeda; Travel Expenses: Takeda; M.B.: Advisory boards: Bristol Myer Squibb, Janssen, Glaxo Smith Kline, Pfizer, Sanofi, Stemline Takeda, Menarini Stemline; Speakers Bureau: Sanofi, Takeda, Menarini Stemline, Pfizer, Bristol Myers Squibb, Glaxo Smith Kline; Research Support: Janssen; N.L.: Stock ownership: AbbVie, Checkpoint Therapeutics; Research Support: Omeros Pharma Corporation; L.A.: Speaker Bureau: EUSA Pharma, Novartis, Kite, Beigene; Data safety monitoring board/Advisory board: Roche, Janssen‐Cilag, Verastem, Incyte, EUSA Pharma, Celgene/Bristol‐Myers Squibb, Kite/Gilead, ADC Therapeutics, Novartis; Travel and meeting support: Roche; S.M.: Advisory board: Janssen, Sanofi, Pfizer, GSK; Honoraria: Bristol‐Myers Squibb, Pfizer, Sanofi, AMGEN, GSK, Janssen, Menarini Stem Line; F.B.: Consultancy/advisory boards: Attralus, Novartis, Astra Zeneca; Lecture fees: Amgen, Janssen, GSK, Lilly, Sanofi; F.G.: Advisory board/Honoraria: Janseen, Takeda, Pfizer, Amgen, Sanofi, Cegene/BMS, Roche; Advisory board: Regeneron, Kite, Astrazeneca; N.K.: Advisory board: Calliditas; J.D.L.R.: Advisory boards: BMS, GSK, Janssen, Pfizer, Speakers Bureau: BMS, GSK, Janssen, Menarini/Stemline, Oncopeptides, Pfizer, Sanofi; E.K.: Honoraria and/or Research Support: GSK, Pfizer, Janssen, Sanofi; All other authors have no conflicts of interest to report.

## Supporting information


**Table S1:** Number of patients submitted and included by participating centers.
**Table S2:** (A) Stages of Chronic Kidney Disease. (B) Myeloma response criteria as defined by the IMWG.
**Table S3:** Validation of IMWG Renal Response criteria using ROC‐analysis for survival prediction at 1 year, *R* ^2^ and C‐index at baseline, 3‐ and 10‐month landmarks.
**Figure S1:** Overall survival in patients diagnosed by renal biopsy or on clinical grounds.
**Figure S2:** Flow chart of patient selection.
**Figure S3:** Violin plot showing the stepwise increase in eGFR by treatment cycles.
**Figure S4:** Landmark analysis of (A) myeloma response at 3 months, (B) myeloma response at 10 months, (C) renal response at 3 months and (D) renal response at 10 months.

## Data Availability

In case of reasonable request, please contact Heinz Ludwig at heinz.ludwig@extern.gesundheitsverbund.at.
