# Supplementary material for: Parameters Associated With Renal Recovery and Survival in Myeloma Patients With Acute Renal Failure to Cast Nephropathy
Source: Am J Hematol. 2025 Oct 24;101(1):56–69. doi: 10.1002/ajh.70104 (PMC12669944; doi:10.1002/ajh.70104)
Supplement: Supplementary file 1 — Table S1: Number of patients submitted and included by participating centers. Table S2: (A) Stages of Chronic Kidney Disease. (B) Myeloma response criteria as defined by the IMWG. Table S3: Validation of IMWG Renal Response criteria using ROC‐analysis for survival prediction at 1 year, R 2 and C‐index at baseline, 3‐ and 10‐month landmarks. Figure S1: Overall survival in patients diagnosed by renal biopsy or on clinical grounds. Figure S2: Flow chart of patient selection. Figure S3: Violin plot showing the stepwise increase in eGFR by treatment cycles. Figure S4: Landmark analysis of (A) myeloma response at 3 months, (B) myeloma response at 10 months, (C) renal response at 3 months and (D) renal response at 10 months. [file AJH-101-56-s001.pdf]

**Supplementary Table 1.**

| Center (Data Access Group) | No. of registered patients | No. of included patients |
|---|---|---|
| National and Kapodistrian University of Athens, Dep. Clin. Therapeutics, Plasma Cell Dyscrasia Unit | 367 | 135 |
| Wilhelminen Cancer Research Institute | 100 | 55 |
| Ankara Üniversitesi Akademik Veri Yönetim Sistemi | 53 | 46 |
| Mayo Clinic, Division of Nephrology, Hematology, Department of Laboratory Medicine and Pathology | 36 | 27 |
| Fondazione IRCCS Policlinico San Matteo | 61 | 23 |
| Université de Poitiers, Centre Hospitalier Universitaire, Dep. of Nephrology & Renal Transplantation | 33 | 23 |
| Medical University of Vienna, Clinic of Internal Medicine I | 28 | 15 |
| Imperial College Healthcare NHS Trust | 11 | 9 |
| University of Torino, Division of Hematology, Myeloma Unit | 12 | 8 |
| University of Torino, San Giovanni Hospital, Nephrology and Dialysis Unit | 16 | 7 |
| Boston University Medical Center, Department of Medicine, Renal Section | 3 | 2 |
| Businco Hospital, Department of Hematology | 5 | 2 |
| The Feinstein Institutes for Medical Research | 6 | 1 |
| University of Minnesota, Division of Nephrology and Hypertension | 3 | 1 |
| Cairo Kidney Center | 2 | 0 |
| Hospital La Fe, Valencia | 11 | 0 |

**Supplementary Table 2A.**

| Stage of Kidney Disease | eGFR (mL/min/1.73 m$^2$) | Renal function |
| --- | --- | --- |
| Stage 1 | $\geq$90 | Mild kidney damage, normal renal function |
| Stage 2 | 60-89 | Mild kidney damage, mild reduction in renal function |
| Stage 3a | 45-59 | Mild or moderate kidney damage, mild or moderate reduction in renal function |
| Stage 3b | 30-44 | Moderate or severe kidney damage, moderate or severe reduction in renal function |
| Stage 4 | 15-29 | Severe kidney damage, severe reduction in kidney function |
| Stage 5 | <15 | Severe kidney damage, kidney failure |

**Supplementary Table 2B.**

| Myeloma Response | IMWG Criteria |
|---|---|
| Complete Response (CR) | Negative immunofixation on the serum and urine and disappearance of any soft tissue plasmacytomas and < 5% plasma cells in bone marrow |
| Very Good Partial Response (VGPR) | Serum and urine M-protein detectable by immunofixation but not on electrophoresis or > 90% reduction in serum M-protein plus urine M-protein level < 100 mg/24 h |
| Partial Response (PR) | > 50% reduction of serum M-protein and reduction in 24 hours urinary M-protein by >90% or to < 200 mg/24 h<br><br>If the serum and urine M-protein are unmeasurable,5 a > 50% decrease in the difference between involved and uninvolved FLC levels is required in place of the M-protein criteria<br><br>If serum and urine M-protein are not measurable, and serum free light assay is also not measureable, > 50% reduction in plasma cells is required in place of M-protein, provided baseline bone marrow plasma cell percentage was > 30%<br><br>In addition to the above listed criteria, if present at baseline, a > 50% reduction in the size of soft tissue plasmacytomas is also required |
| Minor Response (MR) | NA |
| Stable Disease (SD) | Not meeting criteria for CR, VGPR, PR, or progressive disease |
| Progressive Disease (PD) | Increase of > 25% from lowest response value in any one or more of the following:<br><br>• Serum M-component and/or (the absolute increase must be > 0.5 g/dL)<br>• Urine M-component and/or (the absolute increase must be > 200 mg/24 h)<br>• Only in patients without measurable serum and urine M-protein levels; the difference between involved and uninvolved FLC levels. The absolute increase must be > 10 mg/dL<br>• Bone marrow plasma cell percentage; the absolute percentage must be > 10%<br>• Definite development of new bone lesions or soft tissue plasmacytomas or definite increase in the size of existing bone lesions or soft tissue plasmacytomas |

**Supplementary Table 3.**

| | ROC (AUC) | | $R^2$ | | C-index | |
|---|---|---|---|---|---|---|
| | Renal Response groups | Best eGFR groups | Renal Response groups | Best eGFR groups | Renal Response groups | Best eGFR groups |
| Baseline | 0.626 | 0.595 | 0.031 | 0.019 | 0.68 | 0.70 |
| Landmark at 3 months | 0.592 | 0.599 | 0.024 | 0.020 | 0.65 | 0.71 |
| Landmark at 10 months | 0.552 | 0.547 | 0.019 | 0.013 | 0.63 | 0.69 |

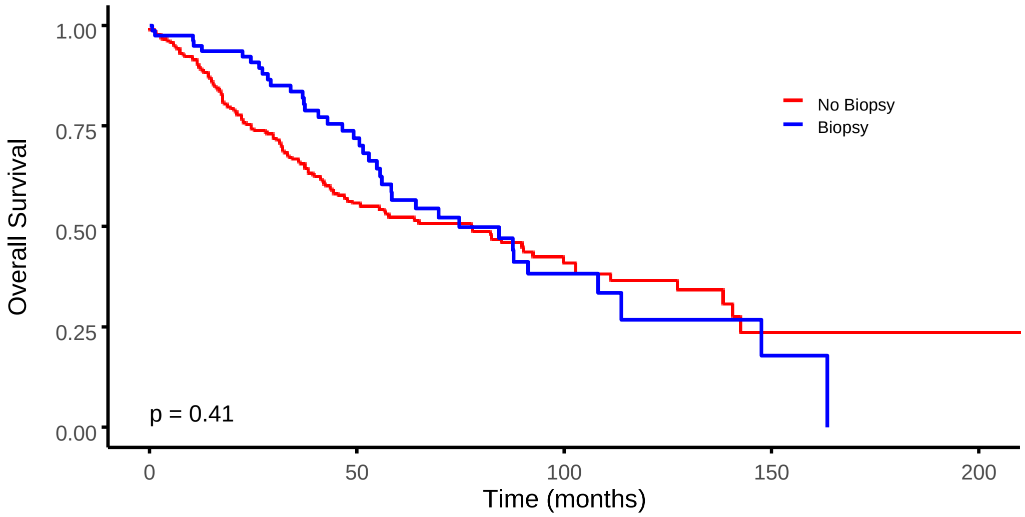


Number at risk

| | | | | | |
|---|---|---|---|---|---|
| No Biopsy (red) | 272 | 87 | 29 | 4 | 1 |
| Biopsy (blue) | 81 | 39 | 11 | 2 | 0 |

Supplementary Figure 1.

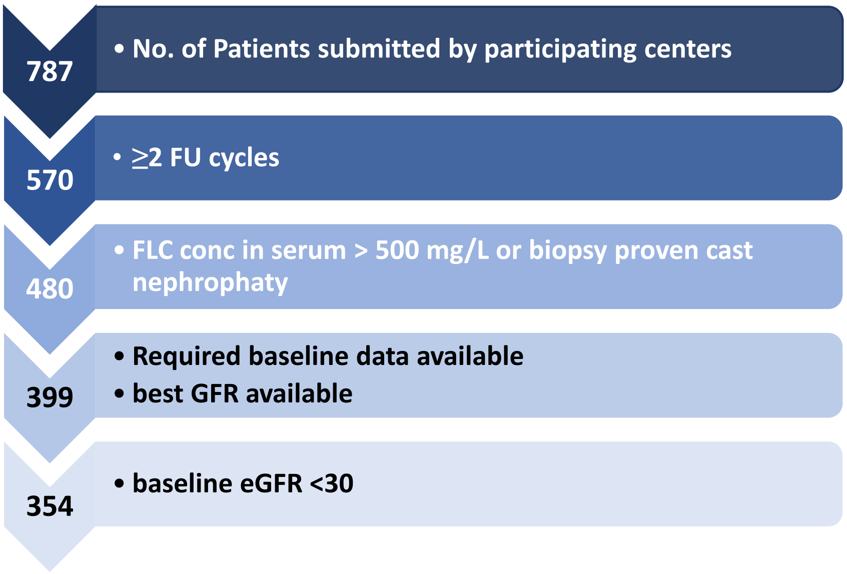


**Supplementary Figure 2.**

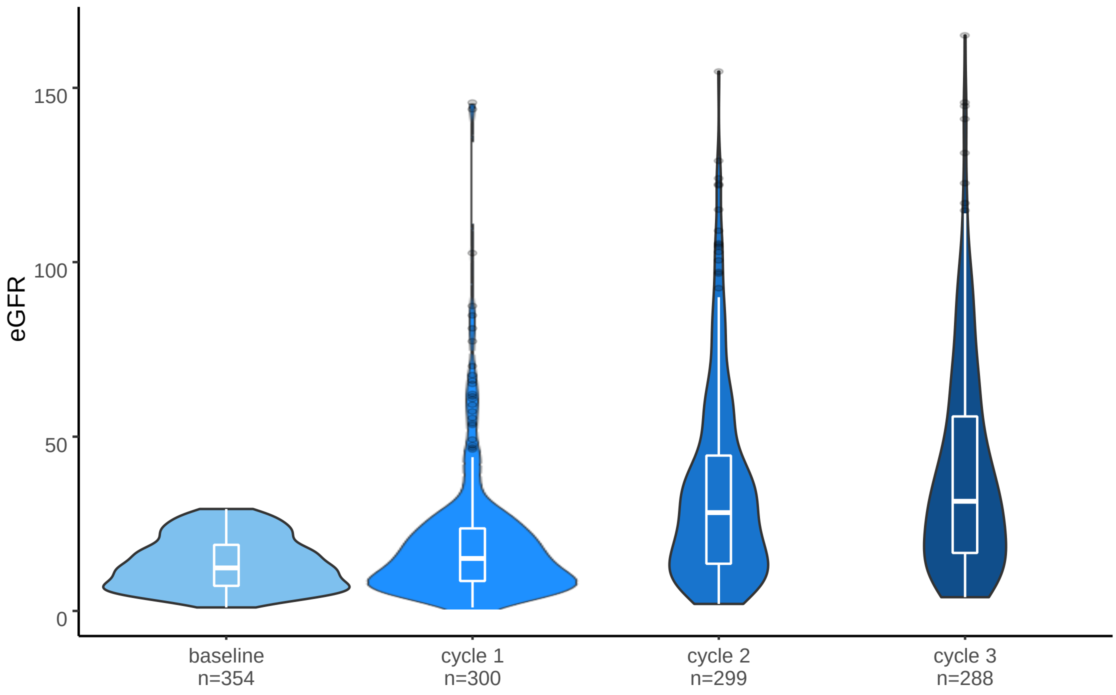


Supplementary Figure 3.

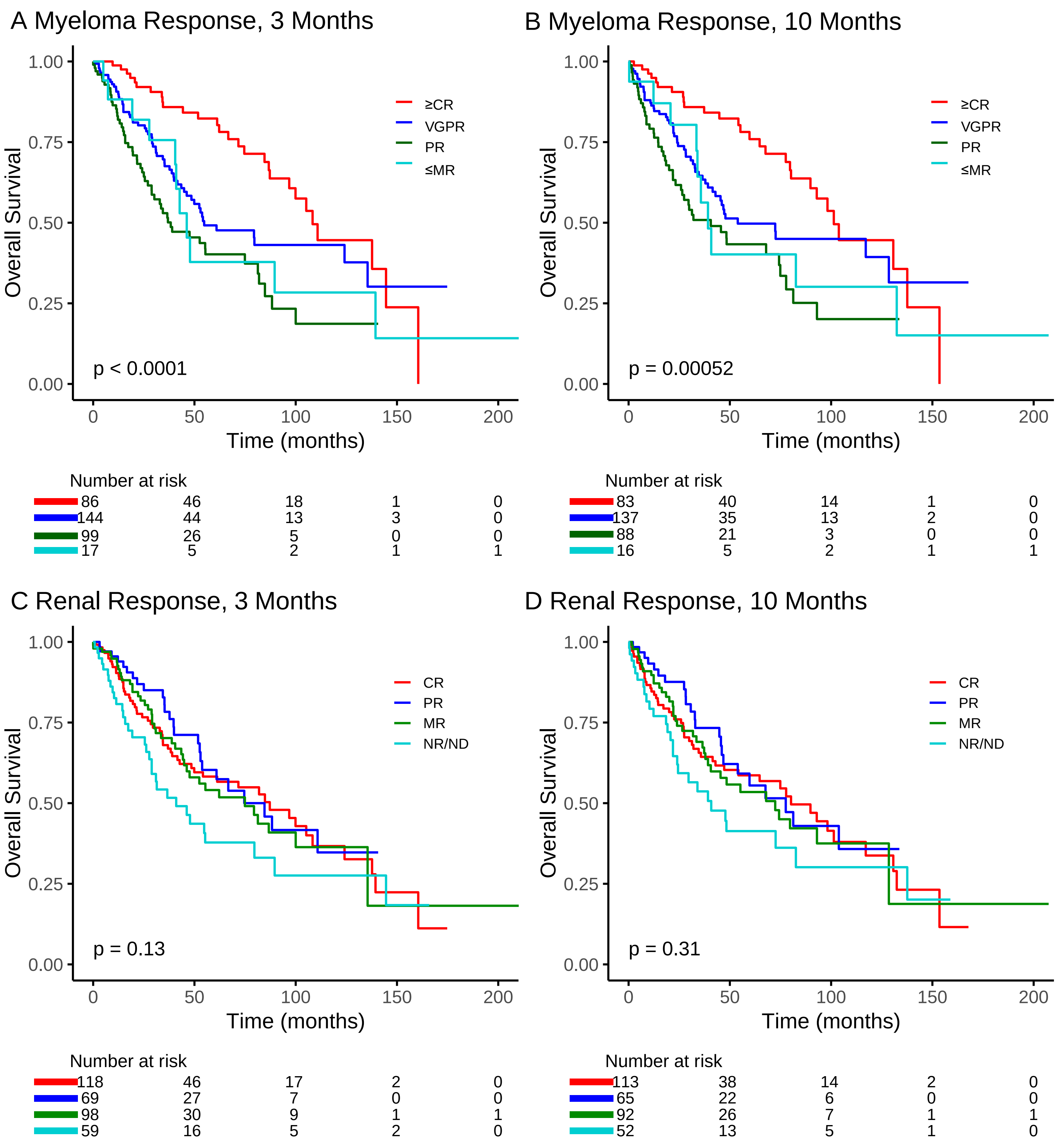


Supplementary Figure 4
